# Vast extension but positive outcomes, reduced but negative: complexity and nuances in evaluating land use by livestock and crops

**DOI:** 10.1093/af/vfae051

**Published:** 2025-04-05

**Authors:** Pablo Manzano, Mariana de Aragão Pereira, Wilhelm Windisch

**Affiliations:** Basque Centre for Climate Change (BC3), Leioa, Spain; Ikerbasque—Basque Foundation of Science, Bilbao, Spain; Embrapa Beef Cattle, Campo Grande, Brazil; Technical University of Munich, Chair of Animal Nutrition, Freising, Germany

**Keywords:** circular economy, food systems, land use, livestock, silvopastoralism

**Implications**

- Livestock uses vast expanses of land but the outcome of such use is often simplistically evaluated. Considering such land use as invariably negative omits the ecological importance of herbivory, mediated either by wild or by domestic herbivores, as well as the factors such as adequate herd composition and management, or adequate governance of grazed lands, which drive to positive outcomes.
- Five broad categories can encompass the totality of livestock systems worldwide. Three of them—extensive grazing, intensive farming reliant on the territory, and backyard systems—are traditional and display high levels of circularity and other positive sustainability traits. Two of them—improved pastures, CAFOs—arose with the large availability of cheap fossil energy that allowed them to achieve very high productivities, but creating challenges regarding sustainability. The five categories often intertwine in a single animal’s life.
- Excessively simplified resource use analysis, such as considering exclusively the mass of main products and co-products, and not their respective economic value, can drive to a flawed identification of factors that drive the shift of food systems towards unsustainability—including both plant-based, animal-sourced, and those that provide both types of foods.
- The role of tree plantations in expanding sustainable land uses has been sometimes misinterpreted by over-simplistic analyses, with many ecologists alerting on possible warming effects via albedo or soil carbon loss. Similarly, the high potential of silvopastoralist systems to produce food while promoting large carbon stocks remains insufficiently explored in spite of sound positive evidence.

## Introduction

Amidst the deep environmental crisis humanity finds itself in, land use change is currently identified as the largest impactor on biodiversity and ecosystem function (Jaureguiberry et al., 2022). While the most impactful and economically profitable land uses at small territorial scales are related to urbanization or mining, they affect a small portion of the global terrestrial surface. It is food production and gathering which uses the largest expanses of land, to the point of having used most of the land for the whole Holocene (Ellis et al., 2021). Food systems are therefore a large focus of the land cover debate.

The global food system replicates the inverse relationship between intensity of impacts. Hunter-gathering is likely to be the food system type with lowest impact—even if having contributed to deep ecosystem transformations due to megafauna extinction (Fricke et al., 2022; Svenning et al., 2024) but also to maintaining landscape structure through controlled burning—yet it has occupied most lands during the longest time, being the dominant use since the dawn of humanity until displaced by more profitable uses a few centuries ago (Ellis et al., 2021). Conversely, croplands are the most destructive (and productive) food production system, yet even in a world of 8 billion people they just occupy 12% of emerged lands. Livestock lies in the middle, being relatively new in humanity’s history but currently being the largest land user. It also has the added complexity of making use of crop products, co-products, and byproducts, and being an enabler to them via fertilizer production and soil restoration in crop rotation.

While it is evident that most land is dedicated to food systems in general, and livestock in particular, research on land use can be affected by excessive simplifications when interpreting sustainability metrics—a problem that is common for other livestock sustainability metrics (Manzano et al., 2023d)—and not appreciating the nuances and trade-offs for each different land use. A paradigmatic example is the views among nature conservationists that advocate for land sparing, confronted with those that pursue land sharing. Maybe the most well-known advocate for land sparing was E.O. Wilson in one of his last works, “Half Earth” (Wilson, 2016), where he advocated for abandoning productive activities in half of our planet, and compensating the loss in food productivity by intensifying uses in the other half. Such extended views trace their roots in cultural conceptions of the Biblical paradise, pristine from human destructive management that followed the original sin. They were reinforced by the first classification of ecosystems by Humboldt (1805), accounting only for forested systems free from human action, and later by the ecological succession theories by Clements (1916) that inevitably saw forests as mature, preserved ecosystems resulting from long-term exclusion of human-mediated disturbances. The cultural impact of such views is evident in some analyses, both in academic papers and in popular science materials such as “Our World in Data,” which consider land use as a homogeneous block, identify livestock as the most important land use problem (https://ourworldindata.org/land-use), and conclude that diets free from animal-sourced foods are the main solution to it (Poore and Nemecek, 2018; Mazac et al., 2022).

By considering the complexity of different land uses and management types, land sharing approaches offer a two-layered analysis: reduced damage with higher long-term sustainability, and land uses with net positive outcomes. Originally, it departed from the perspective that a lesser damage is more sustainable in the long term even if affecting larger portions of land. For land sharing advocates, such lesser damage is preferable to an aggravated damage in more constrained areas, but which will cause major global disruptions because of heightened levels of unsustainability. This is a complex debate, with obvious trade-offs and mixed sparing/sharing proposals (Fischer et al., 2014; Kremen, 2015).

Further complication arises with evidence of some land management types having a positive outcome on biodiversity and ecosystem function when compared with an abandonment scenario (Thompson et al., 2023). The underlying logic of this has been thoroughly developed during the last decades, including paradigm changes in geobotany and vegetation science. For instance, while closed-canopy forests were seen for the last two centuries as the desirable natural ecosystem for most continental areas, it is now considered that alternative nonforested ecosystem states with continuous disturbances such as herbivores would have been the rule and not the exception in biomes where the productivity threshold is below what allows the establishment of rainforests (Bond, 2019; Pausas and Bond, 2020). Domestic herbivores that comply with characteristics such as mobile grazing (Manzano et al., 2023a) can therefore play an important role for ecosystem maintenance, and their disappearance lead to ecosystem deterioration.

Later on, scientists got to understand the crucial role that megaherbivores, such as proboscideans (e.g., elephants) in all continents except Australia, and very large marsupials such as Diprotodon in the latter, had in providing ecological disturbances and mediating ecological processes, whose disappearance would have created a huge gap (Fricke et al., 2022; Svenning et al., 2024). This gap is particularly large for the grazing herbivore functional types (Hofmann, 1989), such as aurochs or wild horses in Europe, who were displaced to extinction by domestic livestock occupying the same niche because of their larger overall ecosystem biomass and therefore their larger productive capacity. The importance of herbivory and megaherbivores is increasingly been acknowledged (Malhi et al., 2022; Pringle et al., 2023; Schmitz et al., 2023) along with the ecological relevance of the open ecosystems they have contributed to maintain, and which should not be lost for afforestations (Bond, 2016; Veldman et al., 2019; Nuñez et al., 2021; Stevens and Bond, 2024). While not that prevalent in the debate, it is known that their role can be fulfilled by grazing livestock if adequate management is provided, notably through adapted governance and through herbivore mobility (Manzano et al., 2023a), especially if considering the large wild herbivore densities that are natural in such settings (Manzano et al., 2023c; Pedersen et al., 2023). Maybe the best example is East African rangelands, where pastoralist milk economies have outcompeted crop farming thanks to a small time gap between dry seasons (Griffith et al., 2020) and have hence managed to maintain a rich wild herbivore fauna (Schieltz and Rubenstein, 2016). Importantly, this does not apply to each terrestrial ecosystem worldwide, but just to those that have a long ecological history with presence of megaherbivores, especially since the radiation of proboscideans from Africa during the mid-Miocene (ca. 15 million years ago), whose disturbances maintain such alternative ecosystem states. Many islands have been spared from such ecological pressure and do show climaxic forests at intermediate levels of ecosystem productivity, as can be shown in the flora of New Zealand, Hawaii or the Macaronesian archipelagos (Canary Islands, Madeira, Açores)—and they also show grave damages from introduced mammalian herbivory (Rubinoff and ‘Ohukani‘ōhi‘a Gon III, 2023). In addition, conserved African large grazing ecosystems, such as the Serengeti-Mara in Tanzania and Kenya (Sinclair et al., 2009), or South Sudan (United Nations 2024), still display wild herbivore guilds with a high diversity and biomass, and a balance composition between herbivore functional types.

Outcomes of livestock on land use are therefore complex, and its understanding requires analyzing how they affect crop and grazing uses, but also how they interact with each other and what the sustainability outcomes are of different management systems. Conventional livestock classifications have focused on the types of feed needed and livestock commodities produced (Steinfeld and Mäki-Hokkonen, 1995) but a thorough analysis of typification according to sustainability outcomes is missing. In the next sections, we provide an overview of the diversity of livestock systems, and their diverse impacts on land use.

## Types of Livestock and Their Outcomes on Land Use—A Typology

In parallel to diversity of land use, a complex gradient of livestock systems can also be observed, with very drastic differences in their outcomes on land use. One dimension of variation is the location of feeding resources they depend on, from systems that largely or completely depend on local natural resources—reliant on their territory, to other systems that are largely or completely sourced from distant resources—giving way to tele-coupled systems. A further dimension is their degree of dependence on grain or crop co-products, with some systems relying exclusively on resources that are neither edible nor cultivated, and others being fully dependent on human-edible crops. Thirdly, there are livestock systems that, under the correct management, have potential to provide rich ecosystem services on their own by efficiently delivering important ecosystem services (grazing and trampling disturbances that facilitate biodiversity conservation, seed dispersal, nutrient recycling or facilitation of pollinator populations), while others will need associated human actions to enable such processes (hay collecting, manure spreading), and even others will trigger environmental damages that undermine such ecosystem services (nutrient pollution, ploughing). Finally, a social dimension presents differences in terms of food sovereignty, i.e., the degree of control that peasants have on their own means of production (see Box 1 in Fischer et al. (2014) for a thorough definition). These elements include considerations on circularity and closeness to, or departure from, wild herbivore ecosystems, that are based on the inherent degree of sustainability (and lack) of a given livestock system and constitute an innovation from former livestock typologies of widespread use, such as e.g., Steinfeld and Mäki-Hokkonen (1995). In this multidimensional setting (Table 1), the following five main types of livestock systems emerge:

**Table 1.** Multidimensional setting that configures the five broad types of farming livestock worldwide

Livestock type	Extensive grazing	Intensive farming reliant on the territory	Backyard systems	Improved pastures	CAFOs
Nutrient path	Circular	Circular	Circular	Linear	Linear
Productivity	Moderate	Low to Moderate	Low to Moderate	Very high	Very high
Food sovereignty	High	High	High	Moderate	Low
Predominant species type	Ruminants	Ruminants	Monogastrics	Ruminants	Monogastrics
Farm management type	Free-range & mobile	Confined	Confined	Free-range	Confined
Attributed climate impacts	High	High	Low	High	Low
Predominant LCA setting	Ecosphere	Ecosphere	Technosphere	Technosphere	Technosphere
Dependence on fossil energy	Low	Low	Low	High	High
Labor demands	High	High	High	Medium	Low
Nutrient pollution	Low	Low	Medium	High	High
Replaceability of natural ecosystem processes	Very high	High	Low	Medium-low	Low

### Extensive grazing

**Figure 1.**
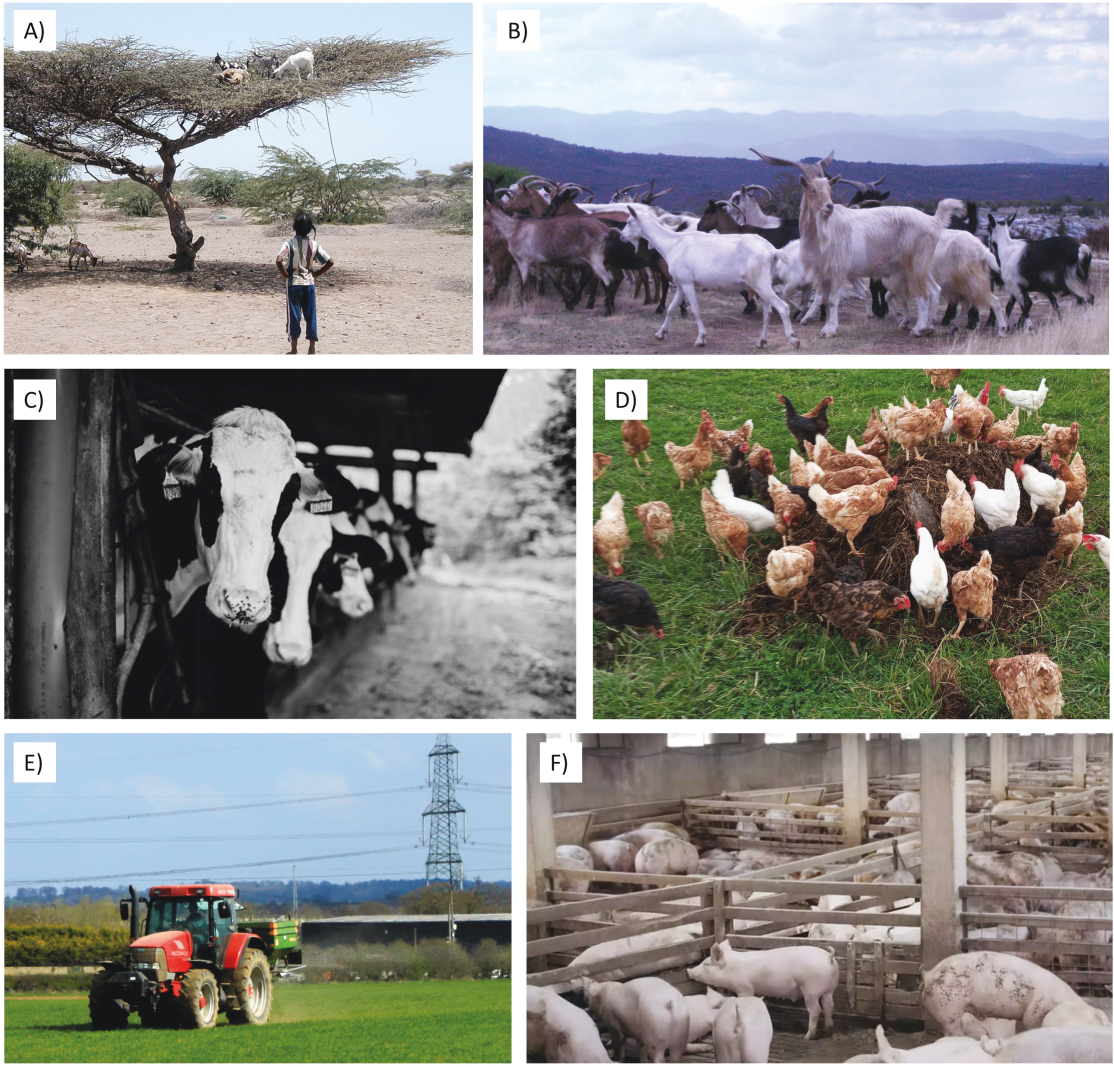
Examples for the five livestock production typologies described. (A) Extensive grazing system in Northern Kenya. Picture: Edmund G. Barrow; (B) Extensive grazing system in Eastern Herzegovina. Picture: Pablo Manzano. (C) Intensive farming reliant on the territory in Northern Spain. Picture: Agustín del Prado; (D) Backyard system in Northern Spain. Picture: Unai Beitia; (E) Improved pastures in Southwest England. Picture: Maurice Pullin/Wikimedia Commons; (F) Pigs in a CAFO. Picture: Dario Sabljak/Shutterstock.

This is a type of system whose feed is based on rangeland resources, including grass but also other cellulose-rich resources, e.g., leaves from woody plants such as tree leaves and shrubs. It comprises a gradient from mobile pastoralist systems that will access fresh, green grass, and leaves according to availability that follows the productivity waves of vegetation (Fig. 1B), to sedentary rotational systems that will administer the standing biomass of pastures for a long time even when they are already dry (Fig. 1A). Seasonally it can feed on crop stubbles, providing organic fertilization in exchange, by creating a rapid remineralization path of the nutrients trapped in plant stems.

Conceptually, such a system applies a localized circular economy scheme, using only local resources that are provided by the natural ecosystem it is nested in, and with very limited anthropogenic inputs from the technosphere (see Figure 1 in Pardo et al. 2024) including fossil-fuel based inputs such as mineral fertilizers or crop products from mechanized systems. Its independence from external inputs usually implies a high degree of food sovereignty. Provided an adequate management is applied (see Thompson et al., 2023; Maree et al., 2025 in this issue), it is also able to furnish a wide array of environmental services that are similar to the ecosystem functions extinct megafauna had—especially on natural rangelands not grazed as fallows. Due to the high proportion of fiber in their diet, such systems have been attributed with a high climatic footprint in terms of methane. This, however, has disregarded that a significant portion of such emissions fall into the ecosphere (Pardo et al., 2024) and therefore do not contribute to anthropogenic climate change. When fully extensive systems with negligible external inputs are compared with natural grazing ecosystems that are conserving their integrity, ecosphere accounts for the practical totality of greenhouse gas (**GHG**) emissions (Manzano et al., 2023b). While their productive capacity is considered to be limited when regarding sedentary systems, the abundance of lands they can make use of and the higher productivity of mobile grazing systems explain the high abundance of herbivores in the past (Pedersen et al., 2023) and the high potential of extensive livestock to take over such niche (Manzano et al., 2023c).

A paradigmatic example is pastoralism with ruminant or equid grazers, evolved in marginal, sparsely populated areas such as drylands, mountains or cold areas with difficult access to markets that could provide inputs. Ruminant browsers can also be important when the dominant fodder resources are woody (Fig. 1A). An emergent type of farm is regenerative grazing that, even if it can access inputs—and may have done so previously in the farm—decides to skip them in the search of a higher environmental sustainability. Large expanses of low-input crop systems have also been traditionally integrated with these livestock systems; here, livestock spends the season where vegetation is growing most (e.g., rainy season, or humid summer) in marginal areas, while it grazes on crop fallows in higher-potential areas during the low-production phase (e.g., dry spell, or winter). In all cases, hardy, often indigenous breeds are used that have a high degree of adaptation to local conditions, but usually with limited high productivity potential.

### Intensive farming, reliant on the territory

Here, livestock is confined in barns (Fig. 1C) but belongs to a small-scale farm that produces most of the fodder inputs on site. Typically, availability of local feed resources varies between seasons with an excess of biomass during the growth season of the vegetation followed by a transient deficiency or even complete absence of feed. The success of these systems strongly relies on techniques to preserve the excess of biomass for the periods of natural feed scarcity, thus enabling the farm to maintain a relatively constant livestock production over the entire year. While hay production through sun drying has been the only viable preservation method for almost the entire history of livestock farming, the development of silage production greatly changed the feed system in middle of the 20th century. It is considerably more effective than sun drying in view of quantity and quality of preserved feed, expands the temporal flexibility of feed preservation, and suits also for biomass grown on cropland (e.g., corn silage). For these reasons, most of intensive farms reliant on territory rely on silage as major feed basis. However, emerging use of photovoltaic and solar heat might lead to a renaissance of hay production, as such preservation techniques may produce excellent feed qualities, and, simultaneously, relieve the farm from the high input of (fossil) energy input for silage production.

Such a system also applies a localized circular economy frame. Nutrients are cycled within the farm, its management adjusted to seasonal availability. Manure, used barn bedding and other residues are reapplied to the farm fields as fertilizer. Traditionally, the inputs from fossil fuel were absent, mechanical energy being provided by draft animals. Contemporary intensive farming that is reliant on the territory uses mostly fossil-fuel mechanical energy but can skip fossil-fuel energy entries for fertilization. Its reduced external inputs also translate into a high level of food sovereignty. Ecosystem disturbances introduced by cultural practices such as hay cutting or farm fertilization imitate to a certain degree the effect of natural herbivores, which translates into e.g., biodiversity maintenance in grasslands or facilitation of pollinator populations. As their imitation departs significantly from what grazing livestock can achieve, their provision of ecosystem services is comparatively limited. Such systems having a diet rich in fiber, they are attributed with a high climatic footprint. Yet in an ideal, purely intensive system reliant on the territory and relying exclusively on local fodder production, with no external inputs, GHG emissions should theoretically not exceed the potential enteric ecosphere emissions of those rangelands where such local fodder is growing. Ecosphere emissions should often be high in past and fully abandoned/rewilded scenarios because of the large natural density of herbivores in many ecosystems (Manzano et al., 2023c; Pedersen et al., 2023). The strength of their productive capacity relies in the use of marginal fiber-rich resources, yet suffers from nutrient and dry matter losses in processes used for storage, such as silage.

Paradigmatic farms for this system typically include ruminants that provide higher productivity outputs, such as many milking systems, because of the reduced energy expenditure of animals and the higher quality of fodder provided. Traditional fattening systems used to fit into these systems, but have now been evolved into confined animal feed operations (**CAFOs**) (see below). Due to the relatively high-quality diet, high performance breeds are preferred to hardy traits.

### Backyard systems

Because of cultural and physiological selectivity, human plant-sourced foods produce a high volume of residues of which livestock has always been profiting. Households have traditionally hosted a reduced number of animals for this purpose, but the processing of crop products translates into a significant volume of residues that has created the niche for associated livestock systems, with very similar characteristics and just a difference in scale. Manure or slurry is collected and used as a fertilizer—either for the orchard in the household, or for the crop fields.

As the previous two, this system has a localized circularity logic. Availability of fodder is logically more constant in the smaller scale household level but subjected to seasonality in the cropland scale. The traditionally animal-sourced draft, particularly for transporting fodder and manure, has been substituted by mechanization fed by fossil fuels, but the inputs in this system are still sourced locally and it prevents fossil fuel-based mineral fertilization. Its circularity often makes it a paradigmatic case for food sovereignty advocates. Its value for biodiversity conservation relies rather in its capacity to prevent expansion of crops, by producing food through a more efficient use of available resources, but it provides few or none direct ecosystem services. The reduced scale of its operation prevents disservices such as water pollution. Life Cycle Analyses (**LCAs**) of such systems consider their climate impacts to be limited, both because of the predominant use of monogastrics (Fig. 1D) and the scarcity of fiber in their diets, and because of use of byproducts for feed. Their productive capacity is limited by the quality of their feed and by its availability, often dependant on the presence of well-designed waste classification systems—be it at the household or farm level, or at a larger urban scales.

Due to the low fiber content of the feeds used, this system typically uses monogastric livestock (poultry, pigs) and, sometimes, also small browser ruminants (goats). Similar to the previous case, traditional fattening systems also took advantage of this system, but have shifted to CAFOs. They display a rich heritage of local breeds adapted to feeding resources that are marginal in nature. There are also cases of such systems thriving in large urban agglomerations, feeding on local resources collected from urban organic waste, the best known example of it probably being Cairo (Ibrahim and Elariane, 2018).

The three systems mentioned above belong to the traditional livestock settings, forced into circularity because of lack of cheap energy available before the industrial revolution. The role of traditional livestock is therefore to produce high quality products from low value resources, supporting the systems they are extracted from either through provision of fertilizer or through ecosystem services.

The following two systems are modern in nature, and depart from a localized circularity into some linear characteristics. They have been facilitated by cheap or readily available fertilization and transport—both related to the availability of fossil fuels. This has translated in very high productivities that explain their current success worldwide, but which also contain the clues for its limitations regarding sustainability.

### Improved pastures

Large-scale production of mineral fertilizer, following the invention of the Haber-Bosch process, facilitated the increase of fodder production in grasslands. Especially in those areas where grasslands are productive all year round, it introduced an opportunity for intensification of extensively grazed systems. Animals graze on the fields with a cellulose-based diet, but the herbivore biomass it sustains is significantly higher than in a natural ecosystem (e.g., an average three-fold increase in Britain; Manzano et al., 2023c).

This system departs from circularity and from resources available within the ecosphere (see Pardo et al., in 2024; Figure 1) inasmuch as it increases production through supplementary fertilizer—manure is fertilizing the fields directly after being excreted by animals, but the extra fertilization (Fig. 1E) comes from mineral nitrogen, potassium, and phosphorus—or from manure and slurry, be it surplus from farms whose rangelands are not large enough to absorb their whole production, or from CAFOs (see below) returning slurry to crop systems because of mandates or efficiencies. Further input of plant nutrients may arise from extra feed supplemented to the animals during periods of low growth rates of grass. It has a high dependence from international commodities, particularly fertilizers, its economic viability being greatly impacted through variations in energy prices, which affect farmers’ food sovereignty. The value in the ecological function of its animals as grazers is greatly reduced by the impoverished biodiversity of pastures compared to a natural rangeland, caused by the dominance of a few, highly nutritious and competitive grass and forb species. The abundance of mineral nutrients often also causes environmental disservices, e.g., pollution of water resources and eutrophication, triggering environmental policy action that has caused unrest among farmers. Due to the dominance of ruminants and fibrous feed resources in such systems, their climate impacts are considered to be high. However, given the higher nutritional quality of fodder species when compared with natural rangelands, they have a smaller attributed footprint per kg of product. The significant departure from baseline grassland production and herbivore biomass in original rangeland ecosystems, as the 3-fold increase in Britain (Manzano et al., 2023c), means that most of their emissions should be attributed to the technosphere and are net contributors to global anthropogenic warming, while only a fraction (a third, in this case) could potentially be attributed to the ecosphere. Such high biomass also explains the very high productivity of these systems.

The grassy nature of these systems causes them to be dominated by ruminant grazers. They originated in areas with plant productivity all year round, with no prolonged dry or cold spells, although genetic improvement of grass and forb species has seen their expansion into more marginal productivity conditions. They are common in mild and oceanic locations of temperate areas, both in the Northern and Southern hemisphere, and spreading in some tropical and subtropical areas, particularly South America. They are normally not integrated with crop systems and use animals that are raised in free-ranging systems, yet with traits selected for high productivity and reduced hardiness.

### Confined animal feed operations

The cheap energy availability that enabled the industrial revolution has brought drastic changes to livestock systems not only in terms of fertilizer availability, but also in terms of cheap transport. Crop farming systems have acquired a scale that goes way beyond the traditional operation of backyard systems. The huge scale of their residues has turned them into co-products that are valued as a commodity in the international market, being transported across vast distances and from one continent to another (Yao et al., 2018). This new availability of fodder multiplied the scale of backyard livestock systems, enabling the establishment of large operations being able to host many thousands of cattle or pigs, or millions of poultry, in a single farm. Manure is not returned into the sourcing crop system because of the cheaper transport option of mineral fertilizer, as it is light in weight because of being devoid of water and has much simpler supply chains, with easy-to-reach economies of scale.

This system departs from circularity in favor of a more linear economy model. Production is not subjected to seasonal variations because it relies on the international commodity market, with a relatively steady supply of fodder that is nevertheless subjected to variations in price—e.g., recent crises motivated by COVID or the Russian war of aggression in Ukraine. The loss of food sovereignty for the farmer does not only root itself in the dependence of distant external outputs, but also on new business models based on vertical integration systems—where, for instance, a company is the owner of the piglets, the farmer fattens them in his or her own facilities (Fig. 1F), and the same company slaughters the finished pigs and processes the meat. In such a system, farmers may become extended work benches or franchise takers by large corporations. Its environmental impacts are, firstly, related to a depart from the ecosphere fully into the technosphere (see Pardo et al., 2024). One implication is, for instance, that their greenhouse gas emissions should be considered as fully anthropogenic. Secondly, the amount and concentration of manure and slurry implies high management costs for its correct application in crop systems. This sometimes causes inadequate or fraudulent disposal that causes pollution of water resources and discomfort of rural communities due to prevalent unpleasant odors. While these environmental problems are evident, the option of not using the residues/co-products of the global industrial crop farming is likely to be even more problematic. CAFOs are often highly efficient feed converters (van Barneveld et al., 2023) that produce high-density nutrient food, and provide an opportunity to generate food that should otherwise be produced by expanding crops, worsening the impacts that crop systems already have on land use.

CAFOs tend to be composed by monogastric livestock (poultry or pigs) due to the more efficient use of high-quality fodder resources, although they are also commonly used in the fattening phase of ruminants. The industrial nature of the facilities hosting them enables locating the farms independently from the local productivity levels in the landscape, but they tend to be located in low-population areas due to the discomfort caused to local communities, and often close to crop, dairy, or food processing plants. They use high-performing breeds that may need controlled temperature and humidity conditions.

The concept of linear economy in CAFOs roots back into the middle of the 20th century, when fertilizers, phytosanitary tools, plant breeding, mechanization, etc. entailed global overproduction of cereals and other high-quality plant materials, which then became available to livestock feeding at virtually unlimited quantities. As an economical consequence, livestock production systems evolved towards maximum efficiency of upgrading these commodities into animal derived food with poultry and pigs being most successful due to their high feed conversion potential. But as soon as growing global population claims cereals, etc. for own consumption, poultry and pigs can hardly adapt towards inferior feed materials because of biological restrictions of their digestive tract. In this situation, the CAFOs can eventually turn from efficient upgraders of high-quality plant materials available in excess into undesirable competitors for human food—although this effect is limited by the low-grade quality of the feed they use, which is mostly not fit for human consumption (van Barneveld et al., 2023).

These five typologies comprise of the main livestock farm types present in the world, but the life cycles of animals often combine phases across several types. Cattle operations in many developed countries typically combine a calving phase on natural pasture in extensive conditions, relying on local resources and with high circularity, with a calf fattening phase at CAFOs (Pereira et al., 2024). The logic behind it is a quick achievement of a live weight that is acceptable for livestock retailers, instead of extending the fattening phase through several seasons that would also include weight loss phases. Such combinations typically make the most of crossbreed strategies, with hardy mothers that are able to adapt to the conditions of marginal pastures, and mixed breed calves that will take advantage of the optimized feeding resources at CAFOs. Such systems typically fall under the widespread term “mixed farming systems” (Steinfeld and Mäki-Hokkonen, 1995), under which umbrella our second typology (“intensive farming, reliant on the territory”) also falls. We deliberately use the term “reliant on the territory” to differentiate the latter from the CAFO typology that makes use of tele-coupled resources and is therefore not reliant on its surrounding territory.

A reverse example of combined farm types in a life cycle is the Iberian acorn-fed pig, whose farrowing phase was traditionally associated with crop areas providing local feed resources, but whose fattening phase takes place in oak woodlands providing abundant acorn feed during autumn and early winter. Pastoralism in Northern Europe, or in many mountain areas where livestock mobility is limited, has also always needed a combination of farm typologies, due to the relatively intense productivity in summer but nil in winter. Here, the opportunity of extensive grazing in summer, usually in more marginal pastures, is combined with hay-making in more high-potential pastures—a hay that is stored for a confined phase in winter. With current low energy prices, many traditionally inspired circular livestock systems incorporate elements of modern linear systems, e.g., semi-extensive grazing systems that incorporate a substantial amount of concentrate feeds in the field (Pereira et al., 2024). Pronounced use of mineral nitrogen fertilizer—an important consumer of fossil energy—further modifies the role of livestock within the entire farming system, as it reduces inclusion of nitrogen and humus collecting plant cultures such as clover into the crop rotation system on arable land. This not only increases the overall harvest of high-quality plant materials suitable for human consumption of CAFOs but also reduces the amounts of nonedible biomass for livestock production exerting circularity. Furthermore, it increases pressure on biodiversity as the cultivation system rotates more quickly with less variety of cultivated plants. In summary, the typologies presented here are a simplification to understand the basic components of what is actually a much more complex reality.

The complexity of farm types and their combinations reflect the complex picture of their outcomes on land use. In general, however, livestock uses marginal resources of the global food system, be it in terms of marginal lands, marginal co-products, or grain resources with less demanding growing requirements and that are not considered edible for humans, and provides a complementarity to the crop system both in terms of resource use—reducing the amount of land to be ploughed, and resource provision—notably in terms of organic fertilizer. The land uses associated with the global livestock system can indeed have a tremendous negative impact on the biosphere, and the demand of CAFOs for co-products can contribute to increasing the economic profitability of crops and to outcompete alternative land uses with higher environmental values, extending the agricultural frontier into areas that would previously have been considered as marginal—South America being the current hotspot in terms of land use change. Yet the reason for such impact is not being a livestock production system, but being part of a global industrial farming system with linear structure and industrial-scale processes based on cheap energy. Global livestock numbers are currently increasing (FAO, 2022), because farms based on a linear economy are expanding. However, circular livestock systems are contracting, which poses a wide variety of problems, e.g., increased wildfires—in parallel to the regression of agroecological crop farming.

## Misallocation of Land Use Change and Its Problems

Oversimplifying the interpretation of land use data and automatically attributing land use impacts is problematic. An example is provided by just attributing most of the expansion of soybean plantations to livestock. Beans from soybean are most commonly processed into soybean oil, as a main product, and soybean cake, as a co-product. Commonly, advocacy materials attribute 80% of such use (and therefore 80% of the land use change responsibility) to livestock consumption (https://wwf.panda.org/discover/our_focus/food_practice/sustainable_production/soy/; https://www.foodnavigator.com/Article/2023/06/14/soy-animal-feed-s-trail-of-deforestation-what-are-the-solutions/; https://healtheplanet.com/100-ways-to-heal-the-planet/soybean-production). Such analyses are based on soybean cake being 81% of the net weight, and soybean oil being just 19% of it (https://tabledebates.org/sites/default/files/2021-12/FCRN%20Building%20Block%20-%20Soy_food%2C%20feed%2C%20and%20land%20use%20change%20%281%29.pdf). However, and even if it is a more complex analytic procedure, the economic allocation procedure also used in Life Cycle Analysis procedures (Pelletier et al., 2015) makes much more sense, for a reason of land use change is the larger profitability of the soybean oil consumption. When taking, e.g., April 2022 as a comparison moment, we see soybean cake to have a mean retailer price of 500 USD per ton (https://www.lgseeds.es/media/Precios-mercado-oleaginosas-15-tabla.pdf), while soybean oil achieved ca. 1,900 USD per ton (https://siip.produccion.gob.bo/noticias/files/123_06052022abdoc_Cotizacion%20de%20precios%2018-22%20abr%202022.pdf). In broad terms it means that, for April 2022, the profit obtained for a kg of beans originates in equal proportion for human- and livestock-related uses. Again, and to further complicate the issue, the increased soybean oil price (https://www.iowafarmbureau.com/Article/Relative-Value-of-Soybean-Meal-and-Soybean-Oil) may originate in the increased awareness among consumers for palm oil impacts on valuable rainforests. Yet possibly, soybean import bans may negatively impact on such rainforests, by increasing the demand for palm oil.

As can be derived by the typology presented here, performance of livestock based on agroecological systems can be positive irrespective of the degree of their intensification. Such positive outcomes can be in terms of land care by adequate grazing by ruminant or equid grazers if they follow basic principles of the productivity-disturbance grazing in ecology, or in terms of reducing ploughed lands by valuating crop and/or food residues and recycling of crop waste, particularly by monogastrics and sometimes also ruminant browsers, thereby preventing the expansion of crop lands. Manure, when applied at adequate concentrations, is a powerful contribution to soil fertility and soil regeneration, and may offer a solution of the challenge humanity faces, with a tremendous dependence on mineral fertilization since the invention of the Haber-Bosch process a hundred years ago, when the world population amounted less than 2 billion humans, and not the current 8 billion. The question arises on whether 6 billion humans are depending on a fossil fuel-based procedure to eat, and how this dependency can be broken. An improved and adequate management of manure in systems with increased circularity is certainly part of the answer.

Grazing and its many different forms and possible ecological and economic outcomes are discussed in more detail in Maree et al. (2025) in this issue, and shall therefore not be elaborated further here. In summary, the question on what makes grazing a positive land use is a hot topic of interest, yet robust underpinnings on grazing ecology rarely make it to land use discussions. Regenerative land uses are known to achieve positive outcomes in terms of biodiversity and carbon stockage (Thompson et al., 2023; Maree et al., 2025 in this issue). On the other hand, grazing is known to bring higher diversity, ecosystem function and carbon storage in higher productivity pastures, but to cause problems in low-productivity ones, at least if only sedentary grazing systems are considered (Maestre et al., 2022; Manzano et al., 2023a, Ren et al., 2024).

De Lange et al. (2025) in this issue is introducing an interactive animal production system tool which aims to resolve the complex dimensions involved when assessing the role of animals in their ecological, nutritional and economic context of a society.

## Expanding Forests: Always Good?

The role of livestock in the land use debate is further misunderstood by an exaggerated role attributed to forests as a climax land cover, as explained in the introduction of this paper. This has at least three undesirable outcomes into the debate: the interpretation of the carbon opportunity cost of keeping landscapes under livestock grazing use, the promotion of tree plantations as a positive land use, and the propagation of forests in biomes where they do not belong, causing biodiversity loss.

The carbon opportunity cost of livestock-grazed landscapes consists of interpreting that many landscapes currently grazed by livestock could potentially be turned into forests through a change into human diets with much lower proportion of animal-sourced foods, with great benefits in terms of increased carbon stocks. Using Hayek et al. (2021) as a typical example of the literature, this interpretation, based on Erb et al. (2018) work on the distribution of natural grasslands and climacic forests, does not account for more modern and widely assumptions in geobotany and vegetation science on savannas, temperate parklands and other Open Ecosystems having a much wider extent than assumed previously, for they form an alternative ecosystem state in areas that host forest if devoid of herbivore or fire disturbances (Bond, 2019). It is these ecosystems that most livestock-grazed landscapes occupy and that naturally accommodate large herbivore populations (Manzano et al., 2023c). Flooding lowlands with large amounts of carbon stocking in the soil are also included in this category, such as the Brazilian Pantanal, areas of the trinational Chaco in Argentina, Bolivia, and Paraguay (Pereira et al., 2024), the Llanos shared by Venezuela and Colombia, or large African wetlands associated with the Nile, Niger, or Zambezi rivers (Manzano et al., 2020). The flora of all these areas has had an evolutionary history of strong herbivore pressure for the last millions of years, decisively changing their tree species to make them different from those belonging to closed-canopy forests (Ratajczak et al., 2017). Such tree types burn more easily. Adding to the pervasive presence of fires in Open Ecosystems, it is not surprising that the carbon stocked in the aerial parts of the vegetation, such as leaves and branches, is not as permanent or reliable as the carbon stocked in the soil (Holdo et al., 2009; Dass et al., 2018)—known to be more abundant in grasslands than in forests (IPCC, 2000). The structural complexity of vegetation in natural rangelands on Open Ecosystems, with woodland, shrub, and grassland patches that accommodate broadleave or conifer trees but that are more protected against fire because of their spatial scattering, is also often overseen. Maps on the areas seen to have potential for increased carbon stocking by converting rangelands, described as “permanent pastures”, into what would be a closed-canopy “forest” category at, e.g., Hayek et al (2021, supplementary figure 2), have an almost complete overlap with the maps describing the extent of Open Ecosystems (Bond, 2005; figure 2.3 at Bond, 2019). Policies based on similar assumptions, as in Denmark (https://climate-laws.org/document/the-climate-act_dae7) or the United Kingdom (UK Government, 2021), have raised concerns among rangeland ecologists for misoriented rangeland afforestation initiatives (Briske et al., 2024; Stevens and Bond, 2024). Finally, carbon opportunity costs interpretations disregard the high greenhouse gas emissions by wild herbivores in landscapes where livestock has been abandoned (Manzano et al., 2023b), which will often not reach the magnitude of the livestock that is currently present but are nevertheless not negligible.

Further questions related to issues previously discussed in this paper delve into a separation of fodder-oriented crops and food-oriented crops (Hayek et al., 2021 supplementary figure 1), not accounting for livestock feeding on co-products, or considering increases in carbon stocks on natural grasslands after livestock abandonment (Hayek et al., 2021 supplementary figure 3)—change towards a positive and well-oriented livestock management being effective in increasing grassland carbon stocks (Maree et al this issue).

Tree plantations are also increasingly being used as carbon stocking schemes to offset emissions, e.g., from commercial flights or other types of consumer-oriented offsets. The rationale behind using them is the easy calculation of cubic meters of wood produced in a hectare of tree plantation, and its conversion in tons of carbon, which makes a promising business opportunity for certifiers. Such plantations, however, have been criticized because of introducing a modification of habitat that departs from the original ecosystem configuration, with negative impacts not only on biodiversity but also on radiative forcing via albedo changes (Hasler et al., 2024) that add to the problematics on wildfires (Nuñez et al., 2021) described above. It should be remembered that, due to the alternative ecosystem state character of Open Ecosystems and their ability to switch from forest to rangeland, it is these systems that are carrying the burden of land use change—with direct impacts on livestock production systems (Briske et al., 2024). Only in ecosystems at intermediate productivity, where alternative ecosystem states are possible, but with natural forests due to a weak ecological history of herbivory (e.g., islands such as New Zealand, Macaronesia, or Hawaii), would forest restoration be justified—but always enabling recolonization of indigenous tree species, and not fire-prone alien tree species from savannas (Ratajczak et al., 2017) and other Open Ecosystems. A possibility that remains unexplored is the utilization of some of such natural dryland forests with ecological equivalents of extinct fauna, such as emus that could function as ecological surrogates of moas in New Zealand (Bond et al., 2004) and which have shown potential for commercial farming under extensive grazing (Sales, 2007).

An intermediate alternative is the introduction of trees in grazing areas, particularly, in sandy soils with low-productivity pasture (baseline), under silvopastoral systems which can improve farms yields, food security (Sekaran et al., 2021), climate resilience, profitability, reduce risks through production diversification and potentially provide additional income sources due to carbon farming and environmental services (Rodrigues et al., 2023). In Brazil, for instance, the annual average carbon accumulation is estimated at 30 kg eucalyptus tree^−1^, which is equivalent to sequestrating 110 kg of CO_2_ tree^−1^ year^−1^, neutralizing cattle enteric emissions. This equivalence was the basis for the development of the first trademark “Carbon Neutral Beef” and its protocol, promoting silvopastoral systems that are eligible to certification by third parties (Alves et al., 2017).

The combination of trees and pasture into silvopastoral systems not only provide shade and shelter to herds (Rodrigues et al., 2023), but can also improve the physical, biological, and chemical soil quality, including greater carbon stocks in soil profiles (Silva et al., 2024).

In summary, the positive role of afforestation in lands subjected to grazed uses is highly dependent on the historical land use (baseline) and tree species to be introduced. Such initiatives should be handled with extreme care in order not to cause environmental damage.

## Conclusion

Judging different land use scenarios affected by livestock systems implies an evident complexity that is unfortunately not accounted for neither in many land use modeling exercises nor in popular science materials or policy analyses. There exist important trade-offs between lowering impacts at the expense of increasing land use, or intensifying use for reducing land demand at the expense of critically worsening impacts. It is urgent to incorporate ecological science into the evaluation of such scenarios, in order to know better the effects of different models of grazing, fertilization and also impacts on expansion or contraction of croplands. Oversimplification leading to wrong interpretations also includes ignoring the economic effects of different products and co-products derived from crop production. Finally, it is urgent to also incorporate up-to-date geobotanical knowledge such as the understanding of Open Ecosystems in land use models, to better understand the outcomes of some changes proposed. In summary, land use science and land use change monitoring require to continue applying multidisciplinary analyses and evaluations, as recommended by the Global Land Programme Scientific Steering Committee (2024).

## About the Authors

**Pablo Manzano** works as an Ikerbasque Fellow at the Basque Centre for Climate Change in Bilbao, Spain. A rangeland ecologist, he graduated at the Autonomous University of Madrid and has gathered research experience at the FSU Jena, Germany, and the University of Helsinki, Finland, plus shorter stays at Landcare Research, Christchurch, New Zealand, and Texas State University, San Marcos, USA. In parallel, he has developed a career in international development cooperation, with experience at several NGOs as well as international organizations such as IUCN, FAO, and the World Bank. His experience has allowed him to perform transdisciplinary research on livestock-based socio-ecosystems. **Corresponding author**: pablo.manzano@bc3research.org

**Mariana de Aragão Pereira** has a bachelor in Animal Sciences (UFV, Brazil) with a master’s degree in Applied Economics (UFV, Brazil) and a PhD in Agricultural Management (Lincoln University, New Zealand). She works as an agricultural researcher at Embrapa Beef Cattle, specialized in Economics and Farm management, with an emphasis on beef farming and production costs, integrated crop-livestock-forestry systems (ICLF), decision-making, adoption and impact of technologies. She is the leader of the Research Group on Livestock Production Systems and coordinator of the National Program for Good Agricultural Practices—GAP Beef Cattle. She is a member of the Scientific Council of the World Farmers Organization (WFO) and a representative of South America on the Board of the International Farm Management Association (IFMA).

**Wilhelm Windisch** has been a full professor of Animal Nutrition at the Technical University of Munich (TUM), Germany, since 2010. As an agricultural scientist, he finished his doctorate in 1988 and his habilitation in 1995. Prior to his appointment at TUM, he was a full professor of Animal Nutrition at the University of Natural Resources and Life Sciences Vienna (BOKU), Austria (2002–2010). He studies the nutritional properties of feed materials and principles of biomass transformation in the metabolism of agricultural livestock with particular interest on minerals, functional dietary compounds, including antinutritive substances, and the impact of livestock feeding on food quality, food safety, and environment.
